# Cost-related delay in filling prescriptions and health care ratings among medicare advantage recipients

**DOI:** 10.1097/MD.0000000000016469

**Published:** 2019-08-02

**Authors:** Toral J. Parikh, Christian D. Helfrich, Ana R. Quiñones, Gillian L. Marshall-Fabien, Lena K. Makaroun, Marissa A. Black, Stephen M. Thielke

**Affiliations:** a Department of Gerontology and Geriatric Medicine, University of Washington, Health Services Research and Development, VA Puget Sound Health Care System; b Department of Health Services, University of Washington, Health Services Research and Development, VA Puget Sound Health Care System; c Department of Family Medicine, Oregon Health & Science University; d School of Nursing, University of Washington in Tacoma; e Center for Health Equity Research and Promotion, VA Pittsburgh Healthcare System; f Department of Gerontology and Geriatric Medicine; g Department of Psychiatry and Behavioral Sciences, University of Washington, Geriatric Research Education and Clinical Center, VA Puget Sound Health Care System.

**Keywords:** cost-related nonadherence, medicare, patient ratings, prescription cost

## Abstract

Despite higher health care needs, older adults often have limited and fixed income. Approximately a quarter of them report not filling or delaying prescription medications due to cost (cost-related prescription delay, CRPD). To ascertain the association between CRPD and satisfaction with health care, secondary analysis of the 2012 Consumer Assessment of Healthcare Providers and Systems (CAHPS) Medicare Advantage Survey was performed.

Regression models quantified the association between CRPD and rating of personal doctor, specialist, and overall health care. Models were adjusted for demographic, health-related, and socioeconomic characteristics. 274,996 Medicare Advantage enrollees were mailed the CAHPS survey, of which 101,910 (36.8%) returned a survey that had responses to all the items we analyzed. CRPD was assessed by self-report of delay in filling prescriptions due to cost. Health care ratings were on a 0-10 scale. A score ≤ 5 was considered a poor rating of care.

In unadjusted models, CRPD more than doubled the relative risk (RR) for poor ratings of personal doctor (RR 2.34), specialist (RR 2.14), and overall health care (RR 2.40). Adjusting for demographics and health status slightly reduced the RRs to 1.9, but adjusting for low-income subsidy and lack of insurance for medications did not make a difference.

CRPD is independently associated with poor ratings of medical care, regardless of health, financial or insurance status. Providers might reduce patients’ financial stress and improve patient satisfaction by explicitly discussing prescription cost and incorporating patient priorities when recommending treatments.

## 1 Introduction

Even with health insurance, many patients experience high out of pocket costs and experience practical financial consequences from medication expenditures. As a result, patients may choose to forego or delay purchasing prescription medications, a phenomenon known as “cost-related prescription delay” (CRPD), “cost-related medication underuse,” “cost-related nonadherence,” or “cost-related non-collection”.^[1–11]^

Older adults often have limited and fixed income and are burdened with greater health care costs and prescription medications compared to their younger counterparts. While fewer than 10% of older adults lack insurance for prescription drugs, 32% report CRPD.^[2,12]^ Further categorized by type of coverage, 15% privately insured,^[2]^ 20% with Medicare Part D,^[12]^ 12% within Veteran Affairs,^[2,12]^ 25% with Medicaid,^[2]^ and 37% of Medicare beneficiaries without prescription coverage^[2]^ have reported CRPD. Several predictors of CRPD have been identified including low-income^[3]^, multiple chronic conditions,^[2,12]^ functional limitations^[3]^, poor self-reported mental and physical health,^[1–3,11]^ and the lack of prescription drug coverage.^[4,12]^ CRPD has been found to co-occur with other financial stressors such as food insecurity^[1,13]^ and receipt of low-income subsidy.^[8]^ Furthermore, those who report CRPD are also at risk for subsequent decline in disease control and self-reported health.^[6,14]^

Findings from a number of qualitative research studies suggest that although patients have an interest in discussing medication-related costs with their providers, clinicians often fail to do so.^[7,9,15,16]^ Thus, the presence of CRPD might imply a lack of shared decision making between the patient and provider. One way to determine how well health care matches patients’ interests is by quality ratings.^[17]^ Although patient ratings can be difficult to interpret and may not be the ultimate goal of medicine or public health, they are an important proxy for patient satisfaction.^[17]^ While some predictors of patient satisfaction have been identified, such as self-reported health status, doctor communication, and care coordination,^[18,19]^ it remains unknown how CRPD influences patient ratings of health care.

Our objective was to ascertain if CRPD was associated with patient ratings of health care in a large Medicare sample. We hypothesized that individuals with CRPD would be significantly more likely to give poor ratings of health care compared to those without CRPD. We anticipated that sociodemographic, economic and health-related factors would confound the relationship between CRPD and low ratings.

## 2 Methods

We analyzed data from the 2012 Consumer Assessments of Healthcare Providers and Systems (CAHPS) Survey.^[20]^ This survey assesses Medicare Advantage (MA) recipients’ experiences with health care, both generally and specifically related to access and communication. Every year since 1997, each of the MA programs has surveyed 800 randomly-selected enrollees. Respondents are enrollees of MA private health insurance program. About 30% of all Medicare beneficiaries have been enrolled in an MA program.^[21]^ In 2012, the 12-page core survey was mailed, and there was a follow-up reminder call to those who did not return it. Spanish-language surveys were sent to those who returned a postcard indicating this preference. Of the 274,996 eligible CAHPS participants, 101,910 (36.8%) returned a survey that had responses to all the items we analyzed. The University of Washington Institutional Review Board determined that this project was not human subjects research.

We considered several questionnaire items to delineate the relationship between CRPD and health care ratings. Self-rated health was assessed with a single question: “In general, how would you rate your overall health?” We dichotomized the answer as well (excellent, very good, or good health) or unwell (fair or poor health). Chronic conditions were self-reported and measured by asking the participant if a doctor had ever told them they had 1 or more of the following 6 conditions:

(1) heart attack;
(2) angina or coronary artery disease;
(3) stroke;
(4) cancer other than skin cancer;
(5) emphysema, asthma, or COPD; and
(6) any kind of diabetes or high blood sugar.

This variable was categorized as none, 1, 2, and 3 or more. Insurance coverage for medications was determined by the question, “Do you have insurance that pays part or all of the cost of your prescription medicines?” Income status was assessed by an administrative variable: low-income subsidy, which identified individuals who received Supplemental Security Income.^[8]^

Our outcome domains were patient ratings of their experience with:

(1) personal doctor,
(2) specialist, and
(3) health plan.

Three questions asked participants to rate their personal doctor, specialist provider, and overall health care in the past 6 months. Under the section titled *Your Health Care in the Last 6 Months*, the survey asked, “Using any number from 0 to 10, where 0 is the worst health care possible and 10 is the best health care possible, what number would you use to rate all your health care in the last 6 months?” Under the section titled *Your Personal Doctor* the survey defines, “A personal doctor is the 1 you would see if you need a check-up, want advice about a health problem, or get sick or hurt.” The survey asked, “Using any number from 0 to 10, where 0 is the worst personal doctor possible and 10 is the best personal doctor possible, what number would you use to rate your personal doctor?” Under the section titled *Getting Health Care From Specialists*, the survey defines, “Specialists are doctors like surgeons, heart doctors, allergy doctors, skin doctors, and other doctors who specialize in 1 area of health care.” A subsequent question was, “We want to know your rating of the specialist you saw most often in the last 6 months. Using any number from 0 to 10, where 0 is the worst specialist possible and 10 is the best specialist possible, what number would you use to rate that specialist?”

The distribution of these scores demonstrated that the majority of the respondents gave their personal doctor, specialist doctor, and health care ratings of 6 or more (95.9%, 94.9%, 91.7%). Furthermore, we assumed that satisfied respondents would give their providers and health care a rating a 6 or above (at least more than half of 10). Thus, we defined any score of 5 or less as an indicator of a poor rating. The main predictor was CRPD, as measured by the question, “In the last 6 months, did you delay or not fill a prescription because you felt you could not afford it?” The options were “yes”, “no”, or “My doctor did not prescribe any medicines for me in the last 6 months”. Only respondents who answered “yes” or “no” were included in the analysis.

The association between CRPD and low ratings was quantified using 4 regression models:

(1) unadjusted;
(2) adjusted for demographic (age, sex, race, and education) and health (self-rated health and number of chronic conditions) variables;
(3) adjusted for demographic and health variables, as well as low-income subsidy; and
(4) adjusted for demographic and health variables, low-income subsidy, and lack of insurance coverage for medications.

Age, sex, race, education, self-rated health, and number of chronic conditions were all asked by the CAHPS survey. We determined if the respondent had insurance coverage for medication based on the survey question, “Do you have insurance that pays part or all of the cost of your prescription medicines?” The receipt of low-income subsidy was acquired from administrative data. We report relative risks (RRs)^[10]^ and consider *P* values <.01 to indicate significant association due to multiple comparisons. Stata/MP 15.1 for Windows (64-bit) was used for all analysis.

## 3 Results

Of the 101,910 (36.8%) surveys we analyzed, the average age was 72.8 years, while 10% were aged below 65 years. Fifty-five percent of the respondents were women, 89% reported being White, 53% reported some college education, 85% reported having insurance coverage for prescription medications, and 84% received an income subsidy. (See Table 1.)

**Table 1:**
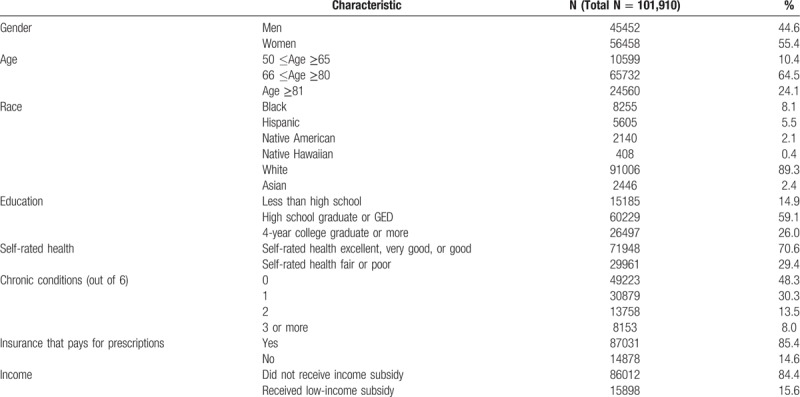
Characteristics of all participants.

The rates of CRPD and low rating of health care are displayed in Table 2. Of all respondents, 13.3% reported CRPD, 4.1% gave a poor rating to their personal doctor, 5.1% gave a poor rating to specialist, and 8.3% gave a poor rating to overall health care. Respondents under the age 65 (*P* <.001), Blacks (*P* <.001), Hispanics (*P* <.001), Native Americans (*P* <.001), Native Hawaiians (*P* = .001), and participants with less than high school education (*P* <.001) were all more likely to report CRPD. CRPD was also considerably more common among participants with poor or fair self-rated health (*P* <.001), with 3 or more chronic conditions (*P* <.001), without insurance coverage for medications (*P* <.001) and who received a low-income subsidy (*P* <.001). Low ratings followed the same trends, but with less pronounced marginal differences than for CRPD.

**Table 2:**
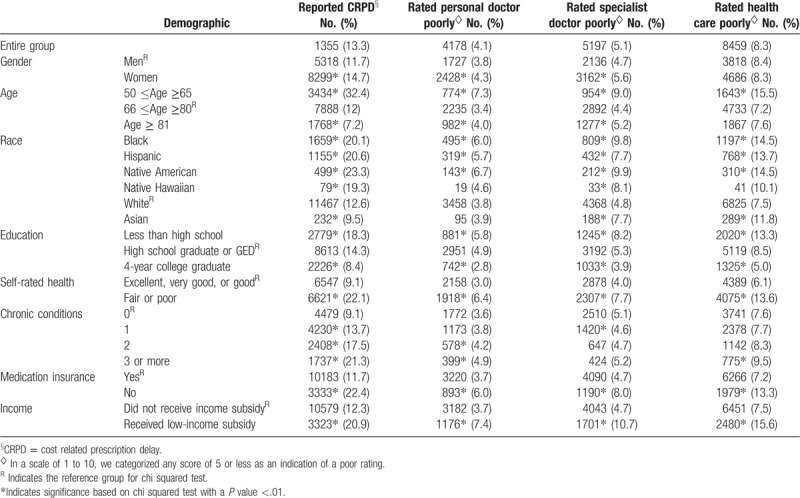
Prevalence of CRPD^§^ and low ratings of medical care based on participant characteristics.

**Figure 1:**
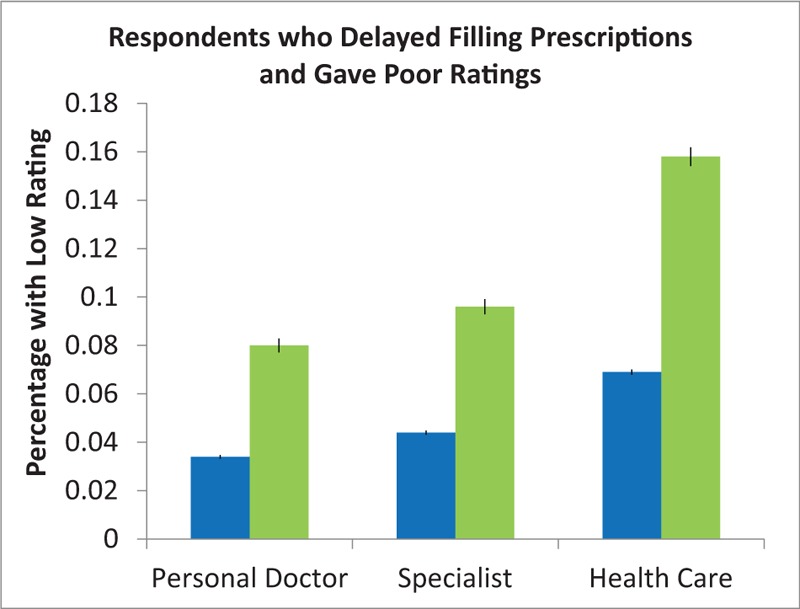
Respondents who delayed filling prescriptions and gave poor ratings. The percent of respondents who delayed filling prescriptions due to cost (green) and gave poor ratings was more than double the percent of those who did not delay filling prescriptions (blue) but also gave poor ratings. Blue bar: Did not delay filling prescriptions due to cost. Green bar: Delayed prescriptions due to cost.

Figure 1 presents the percent of respondents with CRPD who gave poor ratings of their personal doctor, specialist, and overall health care was twice as high as the percent of responds without CRPD who gave poor ratings. Table 3 presents the results of unadjusted and adjusted regression analysis. In the unadjusted model, CRPD more than doubled the RR for a poor rating of personal doctor (RR 2.34, 95% CI 2.18–2.52), specialist (RR 2.14, CI 2.03–2.36), and overall health care in the past 6 months (RR 2.40, CI 2.29–2.53). Adjusting for sociodemographic and health variables slightly lowered the RR to 1.93 (95% CI 1.75–2.13) for personal doctor, 1.91 for specialist (95% CI 1.72–2.11), and 1.95 (95% CI 1.83–2.10) for overall health care and all associations remained statistically significant. Adjusting for low-income subsidy and lack of insurance coverage for medications did not further attenuate the effect size. All *P* values were <.001.

**Table 3:**
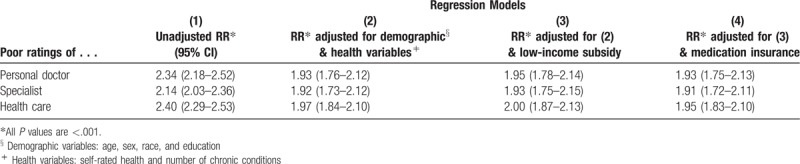
Relative risk for poor ratings of medical care for those who reported cost related prescription delay versus those who did not.

## 4 Discussion

After adjusting for demographic, health-related, insurance-related and income-related variables, CRPD doubled the risk of low ratings of the personal doctor, specialist, and health care in general. In adjusted models, the association between CRPD and ratings was larger than CRPD association with health status, low-income, and insurance for medications. Our results suggest that CRPD is an important and independent determinant of patient satisfaction with care.

Previous studies have inferred that CRPD could be reduced by making prescription drugs more affordable and accessible. However, recent literature has concluded that cultural, interpersonal, and nuanced person-level factors play an important role in CRPD. For example, people with negative medication beliefs and depressive symptoms are more likely to selectively cut back on medications due to cost. ^[3]^ When patients ask their physicians for lower-cost medications, they have a lower likelihood of CRPD, indicating a preference for shared decisionmaking.^[5]^ Many patients would like to discuss medication costs with their providers yet do not do so, and this has been associated with worse disease control and quality of life.^[16]^ Finally, patients who have a trusting relationship with their provider also report lower rates of CRPD and more satisfaction with care.^[3,16]^ In fact, among patients who had higher out of pocket costs, low-income was only associated with cost-related adherence problems in the context of low physician trust.^[7]^ These studies suggest CRPD cannot be simply resolved by making prescriptions more affordable, but may require a multifaceted strategy that improves provider-patient relationship.

While providers are interested in understanding patients’ financial burden and incorporating it into treatment plans, they lack guidance and the opportunity to do so respectfully and comprehensively during busy clinic visits where medical issues often take precedence.^[15]^ Additionally, providers face pressure to follow practice guidelines for managing chronic conditions, but these guidelines generally fail to consider the patients’ economic interests or goals. Insofar as guidelines typically encourage adding rather than removing medications,^[22]^ following them without attention to the patient's experiences might result in decreased patient satisfaction.

Strategies that effectively reduce CRPD by incorporating patient-centered practices would likely increase patient ratings of care. Providers might discuss the patient's goals for medication treatment and the real costs with anticipated benefits of various drug interventions. Shared decision-making frameworks, which elicit and address patient values and preferences, may provide a mechanism to address financial consequences of treatments and reduce CRPD. However, additional research is needed around what “affordable” means to different patients, how to weigh the costs and benefits of treatments, and how to ensure that sufficient discussions about costs occur in clinical settings.

There are a few important limitations to our study. First, our sample was unique in that only one in 8 respondents reported CRPD. This is lower than other studies characterizing CRPD.^[2–4,6,23]^ This could be explained by the fact that majority of our sample had some insurance coverage for prescription drugs, had less comorbidities then the overall Medicare population,^[24,25]^ and that MA enrollees also tend to have slightly higher income than the general population of older adults.^[21]^ Second, the survey included a single question about prescription drugs and does not ask about the total number or type of medications participants were prescribed. Potentially, as the number of medications increases CRPD could also increase, or that certain classes of medication are predictive of CRPD and ratings of health care. Third, our low-income subsidy variable (rather than actual income level) and insurance status for medication (rather than out of pocket costs for medications) may not account for the true relationships between financial stress, prescription medication use, and satisfaction. Similarly, there may be other variables that the CAHPS survey did not capture that may influence the relationship between CRPD and health care ratings. Finally, while the survey response rate was low, which could limit generalizability, other research on CAPHS has not found response bias to significantly skew results.^[26]^

In summary, CRPD doubled the likelihood of poor ratings of providers and overall health care even after adjusting for economic and health-related factors. CRPD may signify patient frustration with healthcare, provider's lack of awareness to the patient's entire life situation, or provision of treatment that is not cost-effective from the patient's perspective. Our results support other published research that providers who discuss economic factors may receive higher ratings from patients. Practice guidelines, which encourage adding medications and generally ignore patient preferences, may contribute to CRPD and thus diminish patient satisfaction. Discussing medication affordability and goals of care might be a straightforward way to improve outcomes and patient experience.

## Author contributions

**Conceptualization:** Toral J. Parikh, Lena K. Makaroun, Marissa A. Black, Stephen M. Thielke.

**Formal analysis:** Toral J. Parikh, Ana R. Quiñones, Gillian L. Marshall-Fabien, Lena K. Makaroun, Marissa A. Black, Stephen M. Thielke.

**Investigation:** Toral J. Parikh, Christian D. Helfrich, Ana R. Quiñones, Gillian L. Marshall-Fabien, Stephen M. Thielke.

**Methodology:** Toral J. Parikh, Christian D. Helfrich, Stephen M. Thielke.

**Supervision:** Christian D. Helfrich, Stephen M. Thielke.

**Visualization:** Toral J. Parikh.

**Writing – original draft:** Toral J. Parikh, Stephen M. Thielke.

**Writing – review & editing:** Toral J. Parikh, Christian D. Helfrich, Ana R. Quiñones, Gillian L. Marshall-Fabien, Lena K. Makaroun, Marissa A. Black, Stephen M. Thielke.
